# Acute Kidney Injury Caused by Obstructive Nephropathy

**DOI:** 10.1155/2020/8846622

**Published:** 2020-11-29

**Authors:** Jonathan S Chávez-Iñiguez, Goretty J Navarro-Gallardo, Ramón Medina-González, Luz Alcantar-Vallin, Guillermo García-García

**Affiliations:** ^1^Servicio de Nefrología, Hospital Civil de Guadalajara Fray Antonio Alcalde, Guadalajara, Jalisco, Mexico; ^2^Universidad de Guadalajara, Centro Universitario de Ciencias de la Salud CUCS, Guadalajara, Jalisco, Mexico

## Abstract

Acute kidney injury secondary to obstructive nephropathy is a frequent event that accounts for 5 to 10% of all acute kidney injury cases and has a great impact on the morbidity and mortality in those affected. The obstruction in the urinary tract has a profound impact on kidney function due to damage produced by ischemic and inflammatory factors that have been associated with intense fibrosis. This pathology is characterized by its effects on the management of fluids, electrolytes, and the acid-base mechanisms by the renal tubule; consequently, metabolic acidosis, hyperkalemia, uremia, and anuria are seen during acute kidney injury due to obstructive nephropathy, and after drainage, polyuria may occur. Acute urine retention is the typical presentation. The diagnosis consists of a complete medical history and should include changes in urinary voiding and urgency and enuresis, history of urinary tract infections, hematuria, renal lithiasis, prior urinary interventions, and constipation. Imaging studies included tomography or ultrasound in which hydronephrosis can be seen. Management includes, in addition to drainage of the obstructed urinary tract system, providing supportive treatment, correcting all the metabolic abnormalities, and initiating renal replacement therapy when required. Although its recovery is in most cases favorable, it seems to be an undervalued event in nephrology and urology. This is because it is mistakenly believed that the resolution and recovery of kidney function is complete once the urinary tract is unobstructed. It can have serious kidney sequelae. In this review, we report the epidemiology, incidence, pathophysiological mechanisms, diagnosis, and treatment of acute kidney injury due to obstructive nephropathy.

## 1. Introduction

Acute kidney injury (AKI) is a complex syndrome that is much more than a simple severity marker. It affects all of the systems, and it can lead to multiorgan failure; thus, it has a profound biological impact in those afflicted by it. Obstructive nephropathy (ON) is a frequent cause of AKI, and when it is acute, it requires emergency medical attention from nephrologists and urologists [1]. ON accounts for 5 to 10% of all AKI cases [2]; however, in the elderly community, it can be present in up to 22% of AKI cases [3]. Due to the nature of their pathologies, patients suffering from urinary tract disorders who attend urology have an increased risk of experiencing AKI, and they are almost always elderly individuals or patients with urinary sepsis [4, 5]. Though AKI caused by ON (AKI-ON) has a more benevolent clinical evolution than other causes of AKI, such as sepsis [6], cardiac surgeries [7], or nephrotoxicity [8], there is, nonetheless, a significant association with prolonged hospital stays, decreased kidney recovery, and a greater chance of death [4], when compared with individuals who do not suffer from AKI-ON. In this review, we assess the etiologies, physiopathological mechanisms, and treatments that result in glomerular and tubular alterations that can lead to AKI-ON.

## 2. Epidemiology

In a cohort study involving patients suffering from urinary tract disorders who attend urology, it was reported that AKI-ON represented 46.5% of urgent-care cases, when quantifying these numbers through AKIN stages, 89.7% were in stage 1, 5.2% in stage 2, and 3.4% were in stage 3. In this study, patients who had undergone emergency procedures had double the AKI incidence rate than patients receiving elective treatments (66.6% vs. 33.4%); urgent-care patients were also older, predominantly male, experienced greater severity of AKI, their 30-day death probability was three times higher (3.6 vs 9.9%), and kidney recovery was less frequent [4]. In another cohort of intensive care (ICU) patients, it was noted that 7.6% of the individuals had AKI-ON [2]. These cases tend to be associated with a malignance, as shown in Table 1; in an additional cohort study involving 49 patients, 83% presented this association, those with AKI-ON had an average life expectancy of approximately 239 days, and 90% of the individuals died within a year [9]. Among patients with AKI-ON, the death rate has been reported to be 20% during their hospital stay, and at 3 and 6 months, it reaches 19 and 28%, respectively; when those numbers are compared with hospitalized patients with AKI but without ON, their respective rates are 8 and 9% [2].

AKI-ON associated to cancer is a very frequent complication, a Danish population-based study reported an 18% incidence of AKI within the first year after a cancer diagnosis, and this combination negatively affected patient survival [10].

When AKI is associated with a cancerous malignancy, it occurs in approximately 10% of the cases of the AKI etiologies in this population [11, 12], and the mortality rate is higher than those without ON [11]. Ureteral obstruction secondary to advanced cancer occurs in prostatic, bladder, and kidney malignancies, and it can also be secondary to extrinsic compression of the urinary tract from both primary and metastatic abdominal or pelvic malignancies [12], as shown in Table 1.

### 2.1. Acute Urine Retention and AKI

Acute urine retention (AUR) can result in the inability to pass urine voluntarily and can lead to ON. The International Continence Society describes this condition as follows: a palpable bladder that is percussible and sensitive to pain when the patient is unable to pass urine [13], it is associated with abdominal pain [14], although this symptom is not always seen, as is the case with patients who have spinal injuries and neuropathies [15]; the AUR incidence rate is estimated to be approximately 3.0 to 6.8 cases per 1,000 individuals/year [16], in younger patients, it can be a side effect of urinary infections or drug side effects [17]; it is more prevalent in adults >65 years, and up to 1 in 3 men who are >80 years will experience AUR at some point in their lives [18]. In Table 2, we describe the AUR etiologies, and these AUR etiologies are divided by intrinsic and extrinsic factors; intrinsic factors are those caused by a direct obstruction of the urinary tract. Postpartum AUR is another example, as it is present in up to 75% of all cases of ON [19], caused by stretching of the urinary system that results from any type of bladder compression [14]. Extrinsic factors are those that result from external causes, such as a mass or a tumor. Among intrinsic factors, benign prostatic hyperplasia (BPH) is the most common cause of AUR in elderly men [20]. Interestingly, there is limited information regarding the epidemiology of AKI-ON. In brief, AUR is the most common clinical picture among patients with AKI-ON; thus, understanding the epidemiology of this association is important for establishing a diagnosis.

## 3. Clinical History and Physical Exploration of Patients with AKI-ON

Clinical history should describe alterations for both emptying and urinary urgency [14], as well as enuresis, history of urinary tract infections, hematuria, renal lithiasis, prior urinary interventions and constipation [1], genitourinary and abdominal neoplasms, and information regarding drug intake (Tables 1 and 2). During physical examination, emphasis should be placed on intravascular volume, and locating an increased vesical volume that usually needs at least 300 mL of urine in the bladder to become palpable [21], a value that is also useful when diagnosing chronic urinary retention, but only when it is measured postmiction [22].

## 4. Effects of ON in Glomerulus

**Figure 1:**
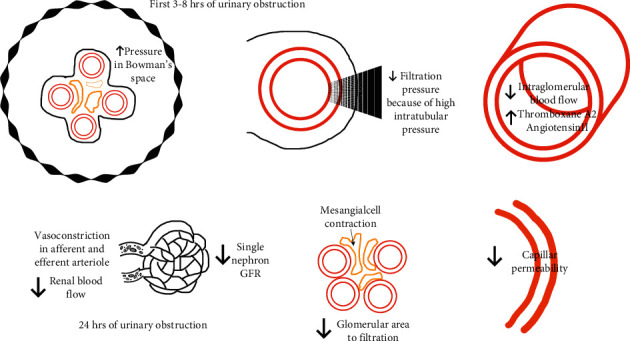
Glomerular changes during ON and AKI. Glomerular changes during ON described according to time in hours. GFR, glomerular filtration rate.

Though glomerular changes are not fully understood during ON, experimental evidence suggests that when it starts (first few hours), the intraluminal pressure transfers to the renal tubules and to Bowman's space, which results in an decreased filtration pressure in the glomerular capillary wall (Figure 1) [23–25]. After 24 hours of ON, the renal and intraglomerular blood flow decrease [23–25] as a result of the intrarenal production of thromboxane A2 and angiotensin II, and these strong vasoconstrictors of the afferent and efferent arteriole (respectively) contribute, in part, to a decreased glomerular filtration rate (GFR); interestingly, it has also been noted that vasoconstrictors decrease the coefficient of ultrafiltration by counteracting the mesangial cells; thus, they decrease the glomerular surface area that is used for filtration. It has been documented that the ability to permeate through the capillary wall also decreases [25].

### 4.1. How ON Affects Renal Plasma Flow and Vasoactive Hormones

After 2-3 hours of obstruction, renal blood flow increases due to prostaglandins [26] and it normalizes within 5 hours, an event that is mediated by the myogenic changes in the afferent artery. After 24–48 hours, the kidney plasma flow decreases up to 60%, as a result of the increase of thromboxane A2 [27].

When obstruction persists, it results in a considerable loss of the tubular brush epithelia, renal vascular rarefaction, and decreased renal blood flow [28].

There are three specific vasoactive systems that decrease the renal blood flow and the GFR; they are the renin-angiotensin system, prostaglandins, thromboxane, and the kinin-kallikrein system [25]; within these systems, there are activation links that can promote an increase in the activity of all of them, promoting endothelial dysfunction (Figure 1).

### 4.2. Alterations in Proximal Tubular Function and in Water and Sodium Reabsorption

**Figure 2:**
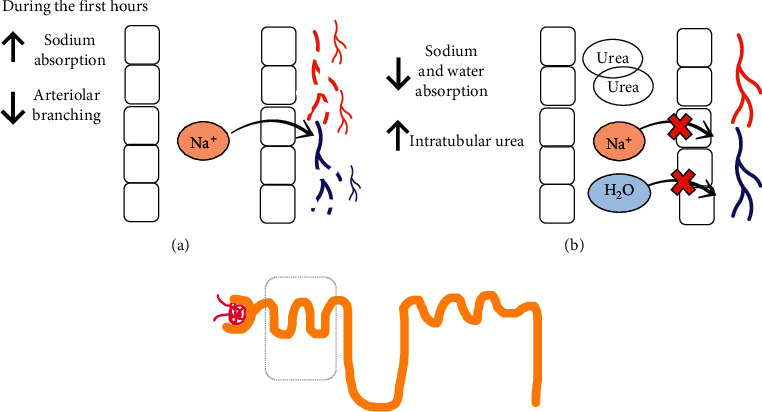
Proximal tubular changes during ON and AKI and after obstruction. (a) Proximal tubule during obstruction. (b) Proximal tubule after obstruction. Functional and structural changes in the proximal tubule during ON and after correction of the obstruction. H_2_O, water; Na^+^, sodium.

During ON, the proximal tubular sodium absorption increases (Figure 2), which is in contrast to a significant decrease in the absorption of sodium in the juxtaglomerular nephrons [29]. The excreted amount of sodium and water increases after the urethral obstruction is cleared, and it decreases the ability to concentrate urine to only 350–400 mOsm [29]; furthermore, as a result of a decrease in medullar tonicity, which is also associated with decreased absorption in the ascending portion of the loop of Henle, there is a notable drop in water absorption in the descending part of the loop of Henle that affects the medulla [30]. An additional factor that interferes with the increase of blood flow in the papillary regions, along with a decrease in TFG in the deep nephrons, results in the depletion of solutes in the medullar interstitium [30]. In mice, this induced severe hydronephrosis and resulted in medullar kidney loss, as well as fluid accumulation in the kidneys; after 8 weeks, there was ipsilateral kidney atrophy, and, in the contralateral kidney, there was compensatory growth. After 1 week with ON, microdissection of the renal arterial trees showed a decrease in arteriolar branching (Figure 2); at 8 weeks, with the persistence of the obstruction, the renal arterial trees revealed extensive damage and tissue atrophy [28].

Collectively, it suggests that the onset of vascular damage may precede the tubular and interstitial damage, and the vasculature characterized by impaired arterial branching and significant arteriolar loss. There is a significant decrease (64–73%) in RBF in ON and could be associated with extensive tubular and interstitial damage [28]. In brief, tubular sodium management during ON can be mediated by tubular alterations in the pump Na-K-ATPasa activity, which results in increased secretion of salt and impairment of urinary acidification. As time with ON increases, it leads to tubular and vascular dysfunction.

## 5. Metabolic Acidosis and Hyperkalemia

**Figure 3:**
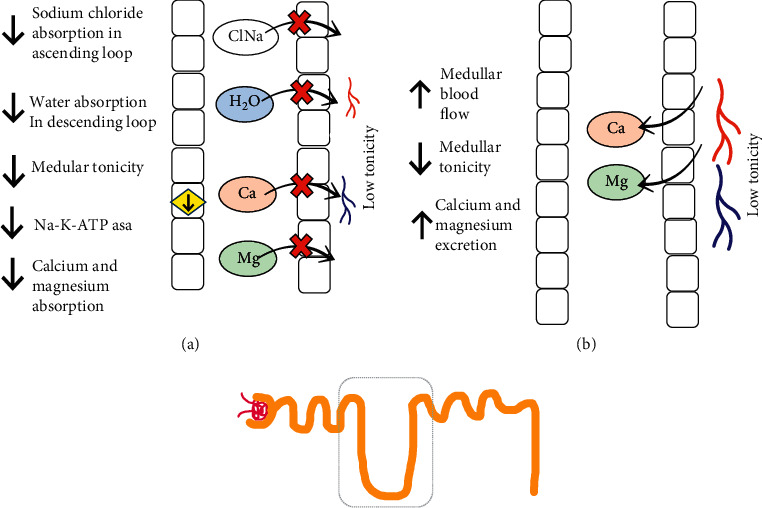
Collecting tubule changes during ON and AKI and after obstruction. (a). Collecting tubule during obstruction. (b) Collecting tubule after obstruction. In the collecting tubules during obstruction, the activity of solute transport pumps, hormonal activity, and the excretion of solutes decrease. After correction of the obstruction, it increases the excretion of free water, K, and hydrogen. Aldo, aldosterone; AMPc, adenosine monophosphate cyclic; AQP2, aquaporin 2; Ca, calcium; NaCl, sodium chloride; H_2_O, water; Mg, magnesium; Na^+^, sodium; PTHr, parathormone receptor; Osm, osmolarity.

Hyperchloremic metabolic acidosis develops as a result of renal acidification inability, partly brought on by the inability to excrete potassium and hydrogen, which is explained by distal renal tubular acidosis, which leads to hyperkalemia despite the adequate or low levels of aldosterone [31, 32] (Figure 3). In interstitial nephropathy caused by ON, hyperreninemia develops, and this limits the production of aldosterone. Another mechanism that explains the alteration of urinary acidification is the mechanism dependent on the voltage, which disables the maximum urinary acidification due to the inability to excrete potassium and the failure to reclaim the sodium [31], which can be identified by its aldosterone deficiency; in the latter, it reserves the ability to lower urinary pH to <5.5 [31], a decrease in ammonium excretion and the inability to increase urinary pCO2 due to poor acid excretion in response to sodium and bicarbonate loads [33–35]. This is especially true with intercalated cells that are responsible for excreting hydrogen (Figure 3). Thus, one of the most important altered cellular mechanisms is believed to be a decrease in epithelial voltage and the deactivation of proton pump activity localized in the luminal membrane [35]. When ON is only present in one kidney, the contralateral also reduces its ability to excrete potassium [36].

### 5.1. Hormonal Alterations during AKI-ON

**Figure 4:**
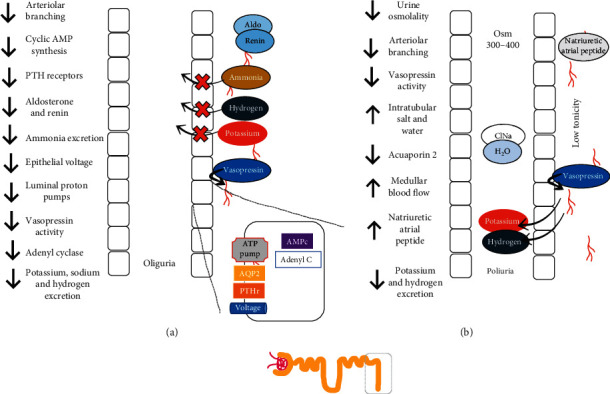
Henle loop changes during ON and AKI and after obstruction. (a) Henle loop during obstruction. (b) Henle loop after obstruction. Essentially, reabsorption, tonicity, and Na-K-ATPase pumps are down during obstruction. After correcting the obstruction, the excretion of Ca and Mg is increased. Ca, calcium; NaCl, sodium chloride; H_2_O, water; Mg, magnesium; Na^+^, sodium.

Vasopressin decreases its action in collecting tubules when the obstruction has been released, and the same thing occurs with the parathormone (PTH). There is also lower synthesis of cyclic AMP and low activation of adenylyl cyclase in basolateral membranes [37], which suggest that these hormonal alterations are secondary to the malfunctioning of secondary messengers (Figures 3 and 4).

### 5.2. Polyuria and Nephrogenic Diabetes Insipidus after Urinary Obstruction Is Cleared

Postobstructive diuresis is a condition generated by the elevated compression of the urinary tract and that compromises the tubular capacity to concentrate urine; it is defined as a urinary output >200 mL, at least 2 hours after decompression, or >3,000 mL in 24 hours [38]. Other explanations are the high amount of urea in renal tubules (caused by AKI), which cause osmotic diuresis [39]. Notably, even low-grade ON can cause alterations in urinary concentrations [40]. The increase in water excretion prior to obstruction reversal is associated with a decrease in juxtaglomerular nephron water absorption and the inability to respond to vasopressin in the cortical collecting tubules [41]. In a study of 62 patients with AKI-ON, it was determined that polyuria occurred in 63% of the cases after clearing an obstruction, this happened 3 hours after to the obstruction reversal, and it lasted for approximately 1.8 days [2], and patients were then able to urinate 7,000 mL. Nephrogenic diabetes insipidus (NDI) develops as a result of renal inability to concentrate urine due to a lack of water reabsorption in the collecting tubule, the clinical picture is with low urinary osmotic concentration, and in some cases, hypernatremia may develop [41]. It is diagnosed through water depravation tests or desmopressin administration, after which, if urinary osmotic concentration is > 800 mOsm/kg, then the results are deemed normal and NDI is ruled out. Urinary osmolarity concentration that is lower than plasmatic concentration is indicative of a deficiency or the absence of aquaporins (AQP2), and it is consistent with NDI (42). NDI is transitory, and it is caused by the direct suppression of AQP2 expression, which is mediated by hydrostatic pressure [41, 42]; it can last up to 30 days after obstruction relief [43].

After an obstruction reversal, the proximal segments absorb more salt and water, but the juxtaglomerular nephrons do not [30] (Figure 3). Another polyuria mechanism is a defect of urinary acidification, which correlates with the inability to absorb sodium and water [35]. In brief, in ON-AKI, there is a decrease in medullar tonicity caused by the inability to reabsorb solutes from the loop of Henle and the decrease of the number of juxtaglomerular nephrons, while increasing the filtration of solutes due to an increased medullar blood flow and a decreased response to vasopressin in the collecting tubule [30]. The retained urea in AKI-ON is excreted prior to obstruction reversal, promoting osmotic diuresis, which also incites water and salt excretion [44]. It has been proven that there is an increase in auricular natriuretic peptide, and this event can play an important role in the excretion of water and salt after obstruction relief; furthermore, potassium excretion increases considerably [45]. Hypercalciuria increases, as does magnesiuria, which can lead to alterations in the reabsorption of solutes in the loop of Henle [46] (Figure 4).

## 6. Diagnosis of Hydronephrosis Caused by ON

**Figure 5:**
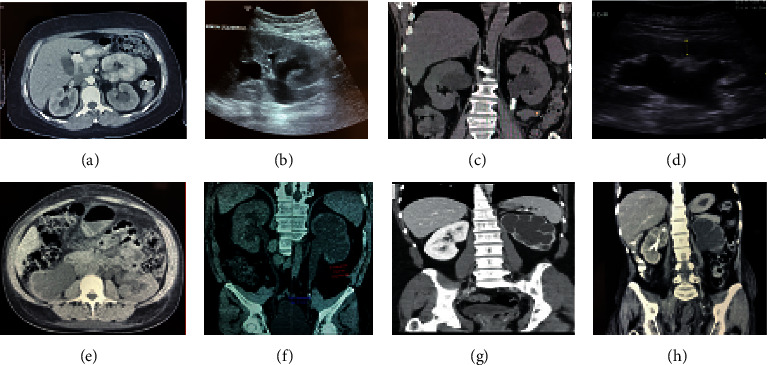
Hydronephrosis grades in ultrasonogram and tomography images. Grade I, slight blunting of calyceal fornices; Grade II, blunting and enlargement of calyceal fornices but easily seen shadow of papillae; Grade III, rounding of calices with obliteration of papillae; Grade IV, extreme calyceal ballooning.

Diagnosis is usually established by the presence of hydronephrosis by either abdominal ultrasonography or computed tomography [47–49] as shown in Figure 5. Hydronephrosis is an anatomical diagnosis, not a functional one, in which there can be caliectasis and pyelectasis without ON. It consists of dilation of the urinary tract caused by multiple urogenital syndromes that culminate in urine retention that expands the upper urinary tract [50], and it increases intrarenal pressure, with progressive dilation of the pelvis and the renal calyx; when it is not corrected, it leads to renal parenchyma atrophy [51]. During the initial stages of ON, hydronephrosis is less evident if there is an intrarenal collective system or dehydration can lead to erroneous interpretations or false negatives. Hydronephrosis is divided into 4 categories, which are arranged in terms of their severity (Figure 5). After 42 hours with ON, changes were observed in ureter pelvic dilation and disappearing of the papilla; after 7 days, pelvic and ureter dilation worsen, as does the weight of the obstructed kidney, parenchyma becomes edematous; on day 12, the renal cortex maintained its augmented size, and there was an observed dilation of the renal calyces; and on days 21–28, the same things occurred with external renal dimensions, there is also diffused thinning of the renal cortex and the medullar tissue. In a porcine specimen, after 6 weeks with ON, kidney size increased, it took on a cystic form, and its weight decreased [52].

When an urgent ON diagnosis is required, “point-of-care ultrasonography” (PoCUS) [51] can be used at the patient's bedside to obtain a quick view of the renal structures [53], and PoCUS also identifies clots, PBH, and hydronephrosis [54]. There is equipment that can rapidly estimate urinary volume [55]. Visualization of the bladder is important when establishing hydronephrosis, if urinary postvoid residual volume is >150 mL, it is suggestive of urinary retention [56]. Kidneys appear to be large, 7–14 cm in adults [57] and 4–10 cm in children [58]. To improve visualization of hydronephrosis, it is recommended to hydrate the patient [59] and to be sure not to cause fluid overload. The dilation of the upper urinary tract tends to appear as a hypoechoic area inside the renal cortex [51, 60] (Figure 5). With the Doppler ultrasonogram, it is possible to evaluate the urethral jets, and the absence or decreased frequency of these implicates urinary obstruction [59]. Recognizing moderate hydronephrosis in the context of renoureteral colic implies there is a 73% chance of a stone of >5 mm [60, 61]. A computed axial tomography (CT) is useful for the recognition of pelvic and abdominal masses. We suggest never delaying a contrast CT for these types of diagnoses, even in the presence of AKI, or any other associated pathology [62, 63]. If spinal compression or cauda equina syndrome is suspected, it is best to use MRI [64]. Estimating and measuring GFR in patients with AKI is not suggested [65]. Though there are considerable limitations, there have been attempts made to evaluate cystatin C in patients with ON, which demonstrates a better performance than creatinine, as its increase is a better indicator of the severity of hydronephrosis [66].

## 7. Treatment

The treatment's aim is to relieve urinary tract obstruction, which alleviates the expansion of the tract and urine accumulation [67], and AKI can be reverted by simply unblocking the urinary tract. Prior to the application of a urinary catheter (UC), benign macroscopic hematuria can appear and tends to revert quickly [68]; at 7 days, it leads to a reduction in hydronephrosis and an improvement in renal morphology [28]. If the patient has a history of stenosis or prostatic diseases, it can be difficult to introduce the UC in which case, the use of 18–20 caliber sizes are recommended [69], and in the event of macroscopic hematuria or an abundance of clots, use a 3 way UC that allows bladder irrigation [70]. It has been proven that in patients with PBH, the early removal of a UC (<3 days) increases the probability of spontaneous urinary voiding, in contrast to leaving the UC in for > 1 week [71]. Thus, we suggest removing the UC as soon as possible. Suprapubic catheterization is reserved for when the application of a UC is unsuccessful [72]. Percutaneous nephrostomy is a well-established interventional method that is performed to divert urine from ON as a temporary treatment [73]; it improves renal function but is associated with significant morbidity, thus affecting quality of life [74]; however, it can also save lives [75]. In 140 patients with ON that had nephrostomy to ON secondary to terminal cancer, the average survival rate was 96 days, with survival in one month, 6 months, and 1 year of 78, 30, and 12%, respectively; the authors of this study used time-point prediction models to estimate the survival rate, which included the presence of 3 or more metastases, the level of hydronephrosis (stage/level 1 or 2) and the serum albumin (<3 g/dL), and patients who only had 1 variable had a 69% chance of 6-month survival, those had 2 variables had a 24% survival rate, and those with 3 variables had a 2% survival rate (74). In a cohort study, this time-point prediction model was validated, and it was noted that hyponatremia also decreases the survival rate [76]. On the other hand, it has been demonstrated that when ON is caused by a malignancy, managing these cases through observation results in a survival rate that is equivalent to an invasive percutaneous intervention [77], since nephrostomy or retrograde urethral stents have not evolved well [69, 73, 78]. After decompression, alpha-1 receptor blockers are suggested, as the bladder, urethra, and prostate are mediated by these receptors [79]. Currently, there is no medical treatment for ON, but pharmaceutical experiments have been carried out to try and limit AKI progression to chronic kidney disease (CKD); these include hydrogen sulfide, which has proven to hinder fibrosis [80] and angiotensin-converting enzyme inhibitors [25]. In cases where polyuria compromises hemodynamics, urinary output should be replaced with 75% of crystalloids by intravascular infusion.

In patients with hyperkalemia and AKI-ON without anuria, the following interventions must be taken:

  Suspend medications that cause hyperkalemia, such as *β*-blockers, nonsteroidal anti-inflammatory drugs, and mineralocorticoid receptor blockers, and reducing the intake of high-potassium foods
  Administer loop diuretics, if GFR <30 mL/min/1.73 m^2^ and fluid overload [81] and serum potassium >5.5 mEq/L [5]

In case of patients with anuria the following is suggested [5]:

  Solution to enhance the transcellular shift: 50% glucose solution, 25–50 g + 10 units of regular acting insulin (0.1 IU kg/weight maximum 10 IU)
  Administer a *β*-2-adrenergic agonist, albuterol (Salbutamol) 5–20 mg, nebulized, maximum effect takes place 90 min after its administration, reducing potassium levels 1–1.5 meq/L
  Sodium bicarbonate (1 meq/kg) intravenous infusion for 10–15 min
  Stabilize cardiac membrane with calcium gluconate 10% IV if changes are observed in EKG
  Ion exchange resins: sodium polystyrene sulfonate 15–60 g divided into 4 doses, other option is administer rectally, proceed with extreme caution when dealing with dehydrated patients, and those who have intestinal obstructions or metabolic ileus
  If hyperkalemia is not reduced, initiate renal replacement therapy (RRT)

Metabolic acidosis (MA) can occur in up to 42% of critically ill patients, more so, among those with AKI-ON [82]. We suggest the following treatment: start sodium bicarbonate infusion according to the “BICAR-ICU” protocol [83]. The infusion starts with 100 mL of a saline solution at 0.9% plus sodium bicarbonate at 4.2% (approximately 7 vials of 8.9 meq), administer for 30 minutes, then measure arterial blood gases 4 hours postinfusion to assess pH levels, and the infusion must be repeated as needed, until pH > 7.30. This strategy reduces the need of RRT and improves the odds of survival in these patients [83]. Some patients cannot tolerate sodium bicarbonate-based treatments or trisodium citrate, and this intolerance can be considered for initiated RRT [84]. The clinical data that suggest uremic syndrome are nonspecific symptoms [85], and it makes it more difficult to decide on whether RRT should be initiated as a treatment option for some patients.

### 7.1. Renal Replacement Therapy for AKI-ON

After the reversal of ON, AKI and its complications are expected to abate; complications can be severe enough to endanger the patient's life, which, in some occasions, may warrant RRT, relieving of the obstruction sometimes is insufficient to mitigate AKI-associated complications, and RRT is thus indicated. In a cohort study involving 62 patients with AKI-ON, 40% of the individuals required RRT [2]. According to the 2012 KDIGO guidelines, RRT should be started when the following are observed: FO, urgent hydroelectrolytic alterations, or imbalance in acid-base homeostasis. Consider each patient's circumstances, the severity of AKI, and the impact that AKI has in distant organs [86, 87]. The probability of requiring RRT increases when there is a history of CKD. RRT has also been justified when FO and diuresis <500–600 mL per day, if there is progressive pulmonary edema, using these criteria have been reported to be beneficial to patient survival [88].

### 7.2. Renal Function Recovery after AKI-ON Is Corrected

The physiopathological mechanisms of AKI-ON lead to intense vasoconstriction with posterior fibrosis [89]. In dogs, it has been observed that a week after ON, the following year, GFR was 25% lower in the affected kidney, when compared with the contralateral kidney [25]. However, in a cohort study involving 34 patients with AKI-ON in the ICU, it was observed that patients without CKD (21%), after 3 months of being treated, still experienced poor renal function; nevertheless, patients who had urinary voids of >7,000 mL during the first day had a 79% chance of recovering renal function within 3 months [2].

## 8. Conclusions

AKI-ON is a frequent occurrence, and it has a big impact on patient mortality, and it leads to profound alterations of all renal functions, which can result in dysregulation of fluids, electrolytes, and acid-base homeostasis; these complications can be harmful to renal function, and they can be potentially fatal. A precise diagnosis requires a thorough clinical history and a full physical examination, and the treatment consists of decompression of the urinary tract and alleviating the metabolic complications that may have been caused by AKI-ON. A complete renal recovery is attainable for some patients, but for others, it can lead to the loss of renal function.

## Figures and Tables

**Table 1:** Causes of obstructive AKI.

Posterior to the bladder	Benign prostatic hyperplasia, cancer, stretching, clotting, acute urinary retention

Ureter	Bilateral obstruction (or unilateral obstruction in patients with a solitary kidney) caused by kidney stones, cancer, or retroperitoneal fibrosis

Renal pelvis	Papillary necrosis, kidney stones

**Table 2:** Etiologies of acute urinary retention (AUR) based on location.

Intrinsic	Kidney stones, malignancies (vesical, prostate), clots, BPH, abscess, stenosis, ureter diverticulum, ureter valves, phimosis, paraphimosis, neurogenic bladder, urinary catheter obstruction, drugs (anticholinergics, anesthetics, antihistamines, analgesics, benzodiazepines, calcium blockers, antidepressants, antipsychotics, antiarrhythmic agents, sympathomimetics, sympatholytics, and muscle relaxers)

Extrinsic	Abdominal or pelvic malignancies, abscesses, atrophic vaginitis, vulvovaginitis, pelvic organ prolapse, aortic aneurism, pelvic fracture, postpartum, fecalith.

## References

[1] Martin J., Chandler W., Speakman M. (2019). Investigating chronic urinary retention. *BMJ*.

[2] Hamdi A., Hajage D., Van Glabeke E. (2012). Severe post-renal acute kidney injury, post-obstructive diuresis and renal recovery. *BJU International*.

[3] Akposso K., Hertig R. A., AlbertiFlahaut C. A. (2000). Acute renal failure in patients over 80 years old: 25-years’ experience. *Intensive Care Medicine*.

[4] Karras G., Williams S. T., McIntyre C. W., Selby N. M. (2013). Acute kidney injury in urology patients: incidence, causes and outcomes. *Nephro-Urology Monthly*.

[5] Moore P. K., Hsu R. K., Liu K. D. (2018). Management of acute kidney injury: core curriculum 2018. *American Journal of Kidney Diseases*.

[6] Ronco C. M. D., Bellomo R. M. D., Kellum J. A. (2019). Acute kidney injury. The Lancet, 394, 1949–1964 Poston JT, Koyner JL. Sepsis associated acute kidney injury. *BMJ*.

[7] Vandenberghe W., Gevaert S., Kellum J. A. (2016). Acute kidney injury in cardiorenal syndrome type 1 patients: a systematic review and meta-analysis. *Cardiorenal Medicine*.

[8] Crass R. L., Rodvold K. A., Mueller B. A., Pai M. P. (2019). Renal dosing of antibiotics: are we jumping the gun?. *Clinical Infectious Diseases*.

[9] Organ M., Norman R. W. (2011). Acute reversible kidney injury secondary to bilateral ureteric obstruction. *Canadian Urological Association Journal*.

[10] Christiansen C. F., Johansen M. B., Langeberg W. J., Fryzek J. P., Sørensen H. T. (2011). Incidence of acute kidney injury in cancer patients: a Danish population-based cohort study. *European Journal of Internal Medicine*.

[11] Eric P., Cohen M. D. (2015). 1, jean-marie krzesinski, MD, PhD,2 vincent launay-vacher, PharmD, ben sprangers, onco-nephrology: core curriculum 2015. *American Journal of Kidney Diseases*.

[12] Soares M., Salluh J. I. F., Carvalho M. S., Darmon M., Rocco J. R., Spector N. (2006). Prognosis of critically ill patients with cancer and acute renal dysfunction. *Journal of Clinical Oncology*.

[13] Abrams P., Cardozo L., Fall M. (2002). The standardisation of terminology of lower urinary tract function: report from the Standardisation Sub-committee of the International Continence Society. *Neurourology and Urodynamics*.

[14] Billet M., Windsor T. A. (2019). Urinary retention. *Emergency Medicine Clinics of North America*.

[15] Sylvester P. A., Mcloughlin J., Sibley G. N., Dorman P. J., Kabala J., Ormerod I. E. (1995). Neuropathic urinary retention in the absence of neurological signs. *Postgraduate Medical Journal*.

[16] Fong Y. K., Milani S., Djavan B. (2005). Natural history and clinical predictors of clinical progression in benign prostatic hyperplasia. *Current Opinion in Urology*.

[17] Mevcha A., Drake M. J. (2010). Etiology and management of urinary retention in women. *Indian Journal of Urology: IJU: Journal of the Urological Society of India*.

[18] Jacobsen S. J., Jacobson D. J., Girman C. J. (1997). Natural history of prostatism: risk factors for acute urinary retention. *Journal of Urology*.

[19] Mulder F. E. M., Hakvoort R. A., Schoffelmeer M. A., Limpens J., Van der Post J. A. M., Roovers J. P. W. R. (2014). Postpartum urinary retention: a systematic review of adverse effects and management. *International Urogynecology Journal*.

[20] Emberton M., Anson K. (1999). Fortnightly review: acute urinary retention in men: an age old problem. *BMJ*.

[21] Abrams P. H., Dunn M., George N. (1978). Urodynamic findings in chronic retention of urine and their relevance to results of surgery. *Bmj*.

[22] Stoffel J. T., Peterson A. C., Sandhu J. S., Suskind A. M., Wei J. T., Lightner D. J. (2017). AUA white paper on nonneurogenic chronic urinary retention: consensus definition, treatment algorithm, and outcome end points. *Journal of Urology*.

[23] Wright F. S. (1982). Effects of urinary tract obstruction on glomerular filtration rate and renal blood flow. *Semin Nephrol*.

[24] Canton A. D., Corradi A., Stanziale R., Maruccio G., Migone L. (1980). Glomerular hemodynamics before and after release of 24-hour bilateral ureteral obstruction. *Kidney International*.

[25] Klahr S., Harris K., Purkerson M. L. (1988). Effects of obstruction on renal functions. *Pediatric Nephrology*.

[26] Moody T. E., Vaughan E. D., Gillenwater J. Y. (1975). Relationship between renal blood flow and ureteral pressure during 18 hours of total unilateral ureteral occlusion. *Invest Urol*.

[27] McGiff J. C., Crowshaw K., Terragno N. A., Linigro A. J. (1970). Release of a prostaglandin-like substance into renal venous blood in response to angiotensin II. *Circulation Research*.

[28] Nagalakshmi V. K., Li M., Shah S. (2018). Changes in cell fate determine the regenerative and functional capacity of the developing kidney before and after release of obstruction. *Clinical Science*.

[29] Buerkert J., Martin D., Head M., Prasad J., Klahr S. (1978). Deep nephron function after release of acute unilateral ureteral obstruction in the young rat. *Journal of Clinical Investigation*.

[30] Klahr P. d. S. (1983). Pathophysiology of obstructive nephropathy. *Kidney International*.

[31] Batlle D. C., Sehy J. T., Roseman M. K., Arruda J. A. L., Kurtzman N. A. (1981). Clinical and pathophysiologic spectrum of acquired distal renal tubular acidosis. *Kidney International*.

[32] Weidmann P., Beretta-Piccoli C., Ziegler W. H., Keusch G., Glück Z., Reubi F. C. (1978). Age versus urinary sodium for judging renin, aldosterone, and catecholamine levels: studies in normal subjects and patients with essential hypertension. *Kidney International*.

[33] Walls J., Buerkert J. E., Purkerson M. L., Klahr S. (1975). Nature of the acidifying defect after the relief of ureteral obstruction. *Kidney International*.

[34] Thirakomen K., Kozlov N., Arruda J., Kurtzman N. (1976). Renal hydrogen ion secretion after release of unilateral ureteral obstruction. *American Journal of Physiology-Legacy Content*.

[35] Sabatini S., Yang B.-L. N. A. (1990). Enzyme activity in obstructive uropathy: basis for salt wastage and the acidification defect. *Kidney International*.

[36] Buerkert J., Martin D., Head M. (1979). Effect of acute ureteral obstruction on terminal collecting duct function in the weanling rat. *American Journal of Physiology-Renal Physiology*.

[37] Stokes T. J., Martin K. J., Klahr S. (1985). Impaired parathyroid hormone receptor-adenylate cyclase system in the postobstructed canine kidney. *Endocrinology*.

[38] Vaughan A. D., Gillenwater J. Y. (1973). Diagnosis, characterization and management of post-obstructive diuresis. *Journal of Urology*.

[39] Narins R. G. (1970). Post-obstructive diuresis: a review. *Journal of the American Geriatrics Society*.

[40] Jaureguiberry G., van’t Hoff W., Mushtaq I. (2011). A patient with polyuria and hydronephrosis: question. *Pediatric Nephrology*.

[41] Bockenhauer D., Bichet D. G. (2015). Pathophysiology, diagnosis and management of nephrogenic diabetes insipidus. *Nature Reviews Nephrology*.

[42] Frokiaer J., Marples D., Knepper M. A., Nielsen S. (1996). Bilateral ureteral obstruction downregulates expression of vasopressin-sensitive AQP-2 water channel in rat kidney. *American Journal of Physiology-Renal Physiology*.

[43] Nielsen S., Frøkiær J., Marples D., Kwon T.-H., Agre P., Knepper M. A. (2002). Aquaporins in the kidney: from molecules to medicine. *Physiological Reviews*.

[44] Purkerson M. L., Klahr S. (1984). Protein intake conditions the diuresis seen after relief of bilateral ureteral obstruction in the rat. *Experimental Biology and Medicine*.

[45] Gillenwater J. Y., Westervelt F. B., Vaughan E. D., Howards S. S. (1975). Renal function after release of chronic unilateral hydronephrosis in man. *Kidney International*.

[46] Davis B. B., Preuss H. G., Murdaugh V. (1975). Hypomagnesemia following the diuresis of post-renal obstruction and renal transplant. *Nephron*.

[47] Benoit D. D., Hoste E. A. (2010). Acute kidney injury in critically ill patients with cancer. *Critical Care Clinics*.

[48] Lau M. W. M., Temperley D. E., Mehta S., Johnson R. J., Barnard R. J., Clarke N. W. (1995). Urinary tract obstruction and nephrostomy drainage in pelvic malignant disease. *British Journal of Urology*.

[49] Lam A. Q., Humphreys B. D. (2012). Onco-nephrology: AKI in the cancer patient. *Clinical Journal of the American Society of Nephrology*.

[50] Krzemień G., Szmigielska A., Bombiński P. (2016). Extreme hydronephrosis due to uretropelvic junction obstruction in infant (case report). *Developmental Period Medicine*.

[51] Cox C., MacDonald S., Henneberry R., Atkinson P. R. (2015). My patient has abdominal and flank pain: identifying renal causes. *Ultrasound*.

[52] Wein A. J. (2016). *Campbell Walsh Urology*.

[53] Rosen C. L., Brown D. F. M., Sagarin M. J., Chang Y., McCabe C. J., Wolfe R. E. (1998). Ultrasonography by emergency physicians in patients with suspected ureteral colic. *The Journal of Emergency Medicine*.

[54] Tsze D. S., Kessler D. O. (2014). Rapid evaluation of urinary retention and penile pain using point-of-care ultrasound. *Pediatric Emergency Care*.

[55] Cooperberg M. R., Chambers S. K., Rutherford T. J., Foster H. E. (2000). Cystic pelvic pathology presenting as falsely elevated postvoid residual urine measured by portable ultrasound bladder scanning: report of 3 cases and review of the literature. *Urology*.

[56] Dicuio M., Pomara G., Menchini Fabris F., Ales V, Dahlstrand C, Morelli G (2005). Measurements of urinary bladder volume: comparison of five ultrasound calculation methods in volunteers. *Archivio Italiano di Urologia, Andrologia: Organo Ufficiale [di] Societa Italiana di Ecografia Urologica e Nefrologica*.

[57] Allan P. L., Allan P. L., Baxter G. M., Weston M. J. (2011). Kidneys: anatomy and technique. *Clinical Ultrasound*.

[58] Rosenbaum D., Korngold E., Teele R. (1984). Sonographic assessment of renal length in normal children. *American Journal of Roentgenology*.

[59] Jandaghi A. B., Falahatkar S., Alizadeh A. (2013). Assessment of ureterovesical jet dynamics in obstructed ureter by urinary stone with color Doppler and duplex Doppler examinations. *Urolithiasis*.

[60] Ellenbogen P., Scheible F., Talner L., Leopold G. (1978). Sensitivity of gray scale ultrasound in detecting urinary tract obstruction. *American Journal of Roentgenology*.

[61] Goertz J. K., Lotterman S. (2010). Can the degree of hydronephrosis on ultrasound predict kidney stone size?. *The American Journal of Emergency Medicine*.

[62] Mohandas R., Dass B., Ejaz A. A. (2019). Contrast-associated acute kidney injury. *The New England Journal of Medicine*.

[63] Vandenberghe W., Hoste E. (2019). Contrast-associated acute kidney injury: does it really exist, and if so, what to do about it?. *F1000Res*.

[64] Mcnamee J., Flynn P., O’Leary S. (2013). Imaging in cauda equina syndrome–a pictorial review. *Ulster Medical Journal*.

[65] Kellum J. A., Lameire N. (2013). Diagnosis, evaluation, and management of acute kidney injury: a KDIGO summary (Part 1). *Critical Care*.

[66] Mao W., Liu S., Wang K. (2020). Cystatin C in evaluating renal function in ureteral calculi hydronephrosis in adults. *Kidney and Blood Pressure Research*.

[67] Bao C.-J., Hsu C.-S., Chen H.-W., Chang C.-H., Tsai P.-C. (2016). Percutaneous nephrostomy versus ureteroscopic management of sepsis associated with ureteral stone impaction: a randomized controlled trial. *Urolithiasis*.

[68] Etafy M., Saleh F., Ortiz-vanderdys C. (2017). Rapid versus gradual bladder decompression in acute urinary retention. *Urology Annals*.

[69] Willette P., Coffield S. (2012). Current trends in the management of difficult urinary catherizations. *Western Journal of Emergency Medicine*.

[70] Jahn P., Beutner K., Langer G. (2012). Types of indwelling urinary catheters for long-term bladder drainage in adults. *The Cochrane Database of Systematic Reviews*.

[71] Djavan B., Shariat S., Omar M. (1998). Does prolonged catheter drainage improve the chance of recovering voluntary voiding after acute urinary retention (AUR)?. *European Urology*.

[72] Goyal N., Goel A., Sankhwar S. (2012). Safe percutaneous suprapubic catheterisation. *The Annals of The Royal College of Surgeons of England*.

[73] Ishioka J., Kageyama Y., Inoue M., Higashi Y., Kihara K. (2008). Prognostic model for predicting survival after palliative urinary diversion for ureteral obstruction: analysis of 140 cases. *Journal of Urology*.

[74] Shekarriz B., Shekarriz H., Upadhyay J. (1999). Outcome of palliative urinary diversion in the treatment of advanced malignancies. *Cancer*.

[75] Wong L.-M., Cleeve L. K., Milner A. D., Pitman A. G. (2007). Malignant ureteral obstruction: outcomes after intervention. Have things changed?. *Journal of Urology*.

[76] Lienert A., Ing A., Mark S. (2009). Prognostic factors in malignant ureteric obstruction. *Bju International*.

[77] Tatenuma T., Tsutsumi S., Yasui M. (2020). Outcome of palliative urinary diversion and observation for malignant extrinsic ureteral obstruction. *Journal of Palliative Medicine*.

[78] Gasparini M., Carroll P., Stoller M. (1991). Palliative percutaneous and endoscopic urinary diversion for malignant ureteral obstruction. *Urology*.

[79] Michel M. C., Vrydag W. (2006). *α*1 -, *α*2 - and *β*-adrenoceptors in the urinary bladder, urethra and prostate. *British Journal of Pharmacology*.

[80] Song K., Wang F., Li Q. (2014). Hydrogen sulfide inhibits the renal fibrosis of obstructive nephropathy. *Kidney International*.

[81] Shen B. F., Clegg D. J. (2019). Physiology and pathophysiology of potassium homeostasis: core curriculum 2019. *American Journal of Kidney Diseases*.

[82] Wesson D. E., Buysse J. M., Bushinsky D. A. (2020). Mechanisms of metabolic acidosis-induced kidney injury in chronic kidney disease. *Journal of the American Society of Nephrology*.

[83] Jaber S., Paugam C., Futier E. (2018). Sodium bicarbonate therapy for patients with severe metabolic acidaemia in the intensive care unit (BICAR-ICU): a multicentre, open-label, randomised controlled, phase 3 trial. *The Lancet*.

[84] Jones S. L., Devonald M. A. J. (2013). How acute kidney injury is investigated and managed in UK intensive care units--a survey of current practice. *Nephrology Dialysis Transplantation*.

[85] Kovesdy C. P., Naseer A., Sumida K. (2018). Abrupt decline in kidney function precipitating initiation of chronic renal replacement therapy. *Kidney International Reports*.

[86] (2012). Kdigo 2012: KDIGO clinical practice guideline for acute kidney injury. *Kidney International*.

[87] Slinin Y., Guo H., Li S. (2014). Provider and care characteristics associated with timing of dialysis initiation. *Clinical Journal of the American Society of Nephrology*.

[88] Chávez-Iñiguez J. S. (2014). Daño renal agudo y manejo de la sobrecarga de volumen. *International Journal of Medical Reviews and Case Reports*.

[89] Chevalier R. L., Forbes B. A. M. S., Thornhill B. A. (2009). Ureteral obstruction as a model of renal interstitial fibrosis and obstructive nephropathy. *Kidney International*.

